# Social networks and internet emotional relationships on mental health and quality of life in students: structural equation modelling

**DOI:** 10.1186/s12888-022-04097-6

**Published:** 2022-07-05

**Authors:** Fatemeh Aliverdi, Hoorvash Farajidana, Zahra Mehdizadeh Tourzani, Leili Salehi, Mostafa Qorbani, Farima Mohamadi, Zohreh Mahmoodi

**Affiliations:** 1 Student Research Committee, Alborz University of Medical Sciences, Karaj, Iran; 2 Department of Emergency Medicine, School of Medicine, Alborz University of Medical Sciences, Karaj, Iran; 3 Department of Midwifery, School of Medicine, Alborz University of Medical Sciences, Karaj, Iran; 4 Department of Health Education and Promotion & Research Center for Health, Safety and Environment, Alborz University of Medical Sciences, Karaj, Iran; 5 Social Determinants of Health Research Center, Shahid Beheshti University of Medical Sciences, Tehran, Iran; 6 Non-Communicable Diseases Research Center, Alborz University of Medical Sciences, Karaj, Iran; 7 Endocrinology and Metabolism Research Center, Endocrinology and Metabolism Clinical Sciences Institute, Tehran University of Medical Sciences, Tehran, Iran; 8 Social Determinants of Health Research Center, Alborz University of Medical Sciences, Karaj, Iran

**Keywords:** Social networks, Internet emotional relationships, Mental health, Quality of life, Students, Path analysis

## Abstract

**Background:**

Social networks and relationships create a sense of belonging and social identity; hence, can affect mental health and the quality of life, especially in young people. The present study was conducted to determine the predicting role of social networks and internet emotional relationships on students’ mental health and quality of life.

**Methods:**

The present cross-sectional study was conducted in 2021 on 350 students at Alborz University of Medical Sciences selected by convenience sampling. Data were collected using five questionnaires: socioeconomic status, social networks, internet emotional relationships, stress, anxiety, depression scale (DASS-21), quality of life, and a checklist of demographic details. Data were analyzed in SPSS-25, PLS-3, and Lisrel-8.8.

**Results:**

According to the path analysis, the DASS-21 score had the most significant positive causal association with internet emotional relationships in the direct path (B = 0.22) and the most negative association with socioeconomic status (B = − 0.09). Quality of life had the highest negative causal association with the DASS-21 score in the direct path (B = − 0.26) and the highest positive association with socioeconomic status in the indirect path (B = 0.02). The mean duration of using social networks (B ≈ − 0.07) and internet emotional relationships (B ≈ − 0.09) had the highest negative association with quality of life.

**Conclusion:**

The use of the internet and virtual networks, internet emotional relationships, and unfavorable socioeconomic status were associated with higher DASS-21 scores and reduced quality of life in the students. Since students are the future of countries, it is necessary for policymakers to further address this group and their concerns.

## Background

The twenty-first century is the era of rapid spread of social networks on the Internet. In contrast to traditional media in which users are passive recipients, social media enables people to create and share content; hence, it has become a popular means of social interaction [1]. Nonetheless, this popularity has led to dramatic changes in people’s lifestyles [2].

According to some reports, 521 million new users have joined social networks by April 2021 [3], and the majority of them are young [2]. Despite people’s increased Internet use and access, the social consequences of its long-term use and associated crises have been neglected [4]. Because of their many similarities to human society, social networks allow people to retain their existing relationships, find new friends and discover information about people they know offline, which can have both positive and negative consequences [5].

Some researchers believe that by creating a sense of belonging and public social identity, social relationships can significantly affect mental and psychological health and improve the quality of life [6]. It has been noted that people with higher social interactions have a more favorable physical and mental health [7]. However, social research and criticisms often emphasize the negative impact of using the Internet [8]. For example, in a study conducted on 1573 young participants, Kim et al. found that internet addiction and using virtual networks are associated with high rates of depression and suicidal ideations [9]. Moreover, some studies observed that the excessive use of virtual networks and the internet are associated with stress, personality disorder, and sleep disorder [10, 11]. In other studies, some researchers reported the negative impact of using the Internet and virtual networks on the quality of life [12, 13]. On the other hand, researchers such as Khalaila et al. (2018) and Schmidt et al. (2021) reported that using the internet has a positive effect on the quality of life [13, 14].

The young population and children are more inclined to use virtual networks due to being alone and not having adequate social connections and support [15, 16]. Robin L et al. (2013) found that being involved in social networks such as Facebook, facilitates access to various forms of support that play a protective role against psychological problems, including stress. It describes several pathways through which participation in social networks can affect psychological well-being [17]. Grino et al. (2017) also found that loneliness affects both mental and physical quality of life [18].

Given these issues and also due to the contradictions existing in relation to the effect of social networks on mental health and social support, the importance of mental health and quality of life among the youth, and the lack of studies assessing all these elements together in one model, the present study was conducted to determine the predicting role of social networks and Internet emotional relationships on quality of life and mental health in students using the structural equation modeling.

## Methods

### Study design and participants

The present cross-sectional study was conducted in 2021 among students at the Alborz University of Medical Sciences in Alborz province and its six schools: medicine, dentistry, pharmacy, health, nursing, and para-medicine.

#### Sample size and sampling method

Based on a study conducted by Veisani et al. [19] and considering ***correlation*** **= 0.15,*β*** **= 0.2,*α*** **= 0.05**, the sample size was determined as 350 using the following formula

$$\boldsymbol{n}={\left\{\left({\boldsymbol{Z}}_{\boldsymbol{\alpha}}+{\boldsymbol{Z}}_{\boldsymbol{\beta}}\right)/\mathbf{C}\right\}}^{\mathbf{2}}+\mathbf{3}$$

$$\mathbf{C}=\mathbf{0.5}\mathbf{In}\left[\left(\mathbf{1}+\mathbf{r}\right)/\left(\mathbf{1}-\mathbf{r}\right)\right]$$

Students were selected via the multistage random sampling method. Students in each school were selected according to the proportional to size method (number of students in each school), and then in each school, students were selected via systematic stratified random sampling method. Stratification was based on the students’ number of passed semesters.

All students aged 18 to 29 years who had passed at least one academic semester, had previous physical and mental health evaluations according to their educational records and self-report, and had a cellphone that enabled them to use virtual networks on their cellphone were included in the study.

The students using psychotropic, narcotic drugs, and antidepressants, who withdrew from the study for whatever reason, who quit studying, experienced adverse events and traumas during this study (e.g., death of their parents), and returned incomplete questionnaires were excluded.

#### Questionnaires

Demographic, socioeconomic status, mental health status, internet use, and quality of life were assessed using validated questionnaires.

Demographic characteristics including age, gender, nationality, marital status, education, the field of study, academic semester, and occupation were asked from students. Students also were asked to report the use of virtual networks on their cellphone, being a virtual network user, type of virtual network used, and the mean duration of using social networks per day (in hours).

#### Socioeconomic status questionnaire

The socioeconomic status questionnaire is comprised of five main items and six demographic items and was developed by Ghodratnama in 2013 to evaluate the four dimensions of the socioeconomic status, i.e., income level, economic class, education, and housing status. The items were scored on a five-point scale ranging from 1: very low to 5: very high (score ranges from 5 to 25). Eslami et al. confirmed this questionnaire’s face and content validity and internal consistency reliability in Iran [20]. Cronbach’s alpha in this study was 0.83.

#### Internet emotional relationships questionnaire

The questionnaire developed by Barghi-Irani et al. [21] was used to assess online emotional relationships in this study. This 28-item questionnaire has five components, including trust, honesty, enjoyment, sexual desire, and preferring virtual relationships, and is scored based on a five-point Likert scale (from totally disagree to agree completely), score ranges from28–140. The validity and reliability of the questionnaire were confirmed with Cronbach’s alpha coefficients of 0.73 for trust, 0.70 for honesty, 0.71 for enjoyment, 0.79 for sexual desire, 0.84 for preferring virtual communication, and 0.90 for the whole scale [21]. In the present study, the questionnaire’s Cronbach’s alpha was 0.85.

#### Stress, anxiety, depression scale (DASS-21)

The validated DASS-21 was used to assess the mental health status of subjects [22]. It has 21 items in the three dimensions of stress, anxiety, and depression, each with seven items, and the final score of each subscale and the total score are found by summing up the scores of the items in each subscale. Each item is given a score between zero (did not apply to me at all) to three (applied to me very much); thus, the total score of each subscale ranges from 0 to 21, and the total DASS-21 score ranges from 0 to 63. The lower total and subscale scores indicate a lower level of depressive and anxiety symptoms and a lower level of stress [22]. The validity and reliability of this scale were confirmed in Iran by Sahebi et al. within a range of 0.77 to 0.79 [23]. The internal consistency of this questionnaire was approved (Cronbach’s alpha: 0.89) in this study.

#### 36-item short form survey (SF-36)

In this study, a Shortened form of health survey called SF-36 was used to assess the quality of life. This questionnaire was designed in the United States by Ware and Sherbourne (1992), and its validity and reliability were assessed in different groups of patients [24]. The survey contains 36 items in eight dimensions, including physical functioning, role limitations due to physical problems, role limitations due to emotional problems, energy/fatigue, emotional well-being, social functioning, pain, and general health. Moreover, two general subscales called physical health and mental health are obtained by combining these subscales. A lower total score in this questionnaire indicates a lower quality of life and vice versa. Score ranges from0–100. In Iran, Montazeri et al. (2005) confirmed the validity of this questionnaire as 0.58 to 0.95 and its reliability as 0.77 to 0.90 [25]. In the present study, Cronbach’s alpha was 0.78.

#### Social networks questionnaire

We used the Iranian questionnaire developed by Jahanbani et al. [26] for assessment. It has 19-items containing three dimensions: the rate of usage, type of use, and the users’ trust in networks. Scoring is based on a five-point Likert scale from very little to very much, score ranges from19–95. Jahanbani et al. confirmed the reliability of this questionnaire with Spearman’s correlation coefficient of 0.90. The internal consistency of this questionnaire was also confirmed with Cronbach’s alpha coefficient of 0.85 [26].

#### Procedure

The study began after obtaining the necessary permissions from the university and a code of ethics from the university ethics committee. Due to the Covid-19 situation and the impossibility of physical presence of the students, a consent form for participation in the study was first sent to the students through related online networks, such as the Student Deputy, the Student Research Committee, and student groups. Eligible students willing to take part were selected by convenience sampling. Then, the online questionnaires were forwarded to the students through these networks, and they were asked to complete them in the specified time frame (maximum of 2 weeks). The researcher’s phone number was given to the students to respond to any possible ambiguities.

The students were assured of the confidentiality of all their data and that they had no obligation to participate in the study or continue their cooperation, and that they would not face any problems or restrictions if they decided not to participate in the study.

#### Statistical analysis

**Fig. 1:**
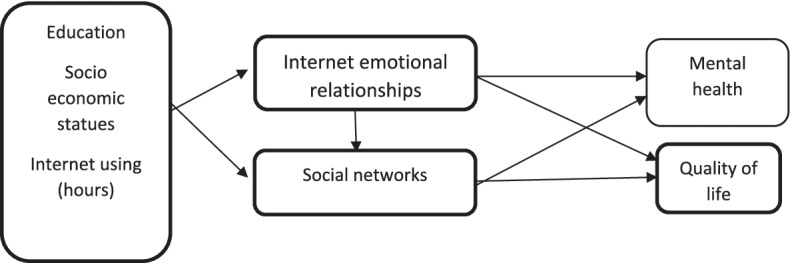
A conceptual model of communication between social networks and Internet emotional relationships on mental health and quality of life

This study assessed the fit of a conceptual model for examining the concurrent effect of social networks and Internet emotional relationships on students’ mental health and quality of life (Fig. 1). First, the normal distribution of the quantitative variables was assessed using the Kolmogorov-Smirnov test, and then the data were analyzed in SPSS-25 [27], PLS3 [28], and Lisrel-8.8 [29].

In order to test the model, the aforementioned questionnaires were first assessed in the model by PLS. The factor loadings of the items of each questionnaire and the validity and reliability of the tools used in the model were assessed in PLS. According to the results, the factor loadings of all the questionnaires items was higher than 0.4 and all the items were retained after the final testing of the model. To determine the convergent and divergent validity, indices including composite reliability (CR), average variance extracted (AVE), maximum shared variance (MSV), and average shared variance (ASV) were used.

The discriminant validity, which means that latent variables that represent different theoretical concepts are statistically different, was assessed by the heterotrait-monotrait ratio of correlations (HTMT); in this measure, values under 0.90 are regarded as having discriminant validity [30].

After assessing the questionnaires in the model we test the model by Path analysis. Path analysis is considered a causal modeling technique; it can be performed with either cross-sectional or longitudinal data and is an extension of the usual regression that shows the direct effects as well as indirect effects and impact of each variable on the dependent variables. All variables in a path model can be described as either endogenous or exogenous. Endogenous variables are diagrammed as being influenced by other variables in the model. The variables diagrammed as independent of any influence are the exogenous variables. Dependent variables are always endogenous**,** but some independent (or predictor) variables can be endogenous if influenced by other independent variables in the model [31]. In this study, exogenous variables were education, internet average time use, socioeconomic status (SES), internet emotional relationships, and the endogenous variable were mental health and quality of life. Social networks were exogenous for mental health and quality of life but endogenous for other variables.

The correlation results were presented as Pearson’s correlation coefficient and the Path analysis results as regression coefficient, Standardized Beta with a significance level of T-value > 1.96.

## Results

The data from 350 students at Alborz University of Medical Sciences were assessed in this study. Participants’ mean age was 22.42 ± 2.80 (range 18 to 29 years), and their mean duration of education was (14.99 ± 1.4 years). The mean score of quality of life was 66.48 ± 15.57 (ranging from 58 to 74), DASS-21 41.31 ± 14.15 (ranging from 21 to 84), internet emotional relationships 61.42 ± 17.50 (range 28 to 99), and social networks 49.87 ± 9.30 (ranging from 26 to 77).

According to Pearson’s correlation test results, the use of social networks (r = − 0.16) and DASS-21 (r = − 0.26) had a negative and significant correlation with the quality of life; with DASS-21 having the highest negative and significant correlation. In other words, the higher the DASS-21 score and its subscales such as stress, anxiety, and depression, the lower the score of quality of life (r = − 0.26) was.

DASS-21 score had a positive and significant correlation with the mean duration of using social networks (r = 0.13) and Internet emotional relationships (r = 0.22). Among these two variables, Internet emotional relationships had the highest positive and significant correlation with the DASS-21 score (r = 0.22) (Table 1). According to PLS analysis, the factor loadings of all the questionnaires items were higher than 0.4, and all the items were retained after the final testing of the model.

**Table 1:** The correlation matrix between social networks and Internet emotional relationships with mental health and quality of life in students 2021(*n* = 350)

		Age	EDU	TUS	INT	DASS	SES	SOM	SFE6
1	Age	1							
2	EDU	0.508**	1						
3	TUS	− 0.108*	− 0.088	1					
4	INT	−0.120*	− 0.134*	0.097	1				
5	DASS	−0.014	0.072	0. 137*	0.222*	1			
6	SES	−0.153**	0.070	−0.156**	0.027	−0.093	1		
7	SOM	−0.085	−0.014	0.497**	0.265**	0.161**	−0.041	1	
8	SF36	0.018	−0.012	−0.056	− 0.074	0.260**	- 0.032	−0.155**	1

** *P* > 0/01 = *P > 0/05

*EDU* Education, *TUS* Average time of use of the Internet, *INT* Internet Emotional Relationships, *SOM* Social Network Score, *DASS* Depression, Anxiety and Stress Scale, *SES* Socioeconomic Status, *SF36* 36-Item Short-Form Survey

In this study, CR > AVE and AVE > 0.5; therefore, the subscales of the questionnaires had convergent validity, and since MSV < AVE and ASV < AVE, the divergent validity was also desirable (Table 2). Furthermore, all variables in this study had discriminant validity. The results of HTMT are shown in Table 3.

**Table 2:** The results of the confirmatory factor analysis and the combination of CR and the mean of variance extracted (AVE) quality of life questionnaire, mental health, social networks and Internet emotional relationships in the test model

Questionnaires	Composite reliability	Average variance extracted	Rho-A	MSV	ASV	Alpha Cronbach
DASS	0.937	0.832	0.904	0.551	0.484	0.899
Internet emotional relationship	0.925	0.712	0.901	0.562	0.389	0.898
Quality of Life (SF36)	0.961	0.74	0.938	0.501	0.435	0.878

**Table 3:** Heterotrait-heteromethod ratio of correlations (HTMT) Matrix Quality of Life, Mental health, Social Networks and Internet emotional relationships in the Test Model

		DASS	INT	SF36	SOM
1	DASS				
2	INT	0.252			
3	SF36	0.750	0.205		
4	SOM	0.209	0.331	0.252	

*INT* Internet Emotional Relationships, *SOM* Social Network Score, *DASS* Depression, Anxiety and Stress Scale, *SF36* 36-Item Short-Form Survey

Values under 0.90 are considered as having discriminant validity

According to the Path analysis, Among the variables that had a significant causal relationship with the DASS-21 score, in the direct path, internet emotional relationships had the most positive significance (B = 0.22), and socioeconomic status had the most negative (B = − 0.09) causal relationship. Moreover, with a one-unit increase in the score of the online emotional relationships, the DASS-21 score increased by 0.22 units, and with a one-unit increase in socioeconomic score, the DASS-21 score was reduced by (− 0.09) units.

**Fig. 2:**
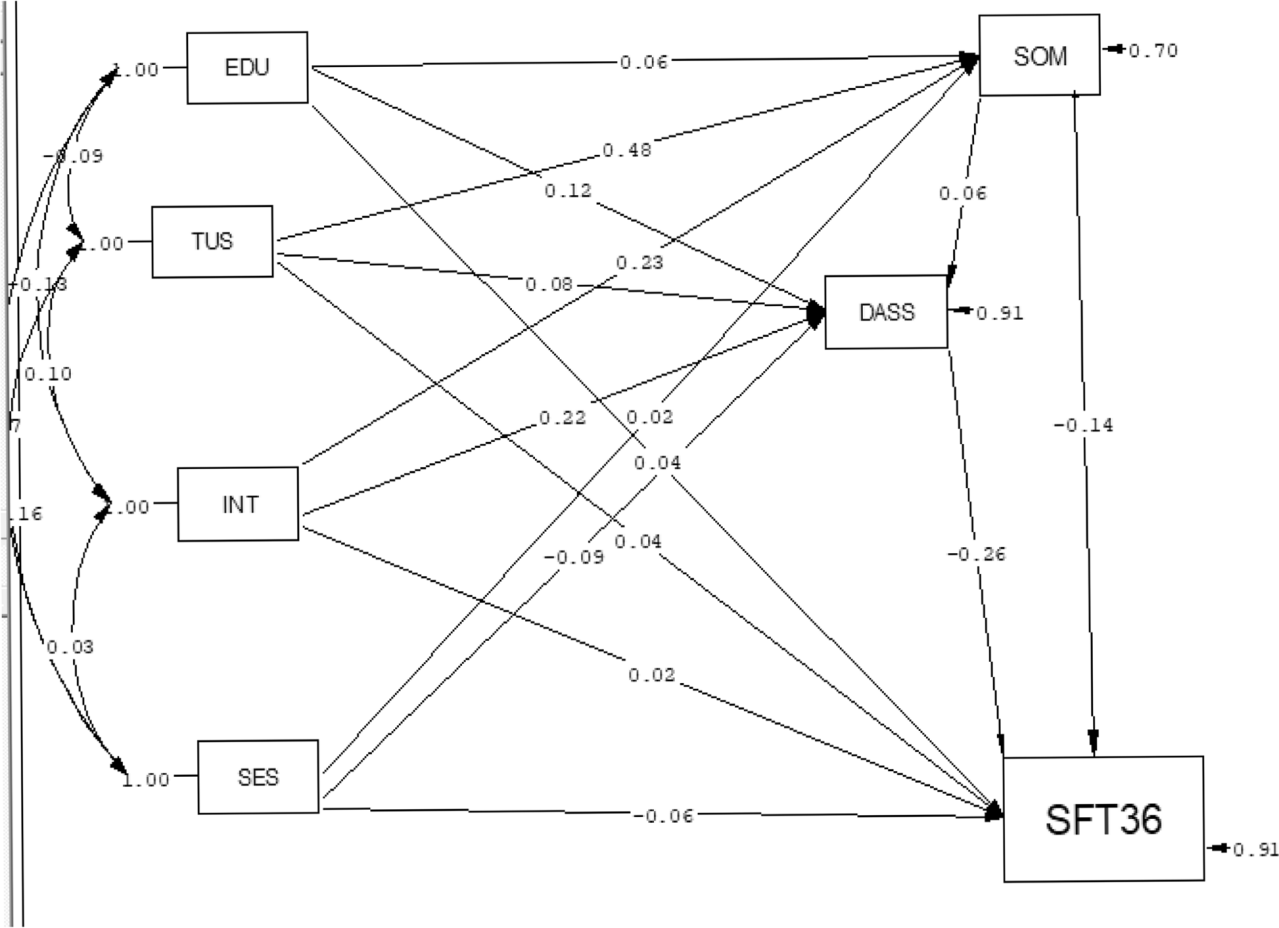
Full Empirical Path Model between Social networks and Internet emotional relationships on mental health(DASS21) and quality of life in students. Single-headed arrow means regression coefficient, Standardized Beta. Two-headed arrow means correlation. EDU = Education. TUS = Average time of use of the Internet, INT = Internet Emotional Relationships. SOM = Social Network Score, DASS = Depression, Anxiety and Stress Scale, SES = Socioeconomic Status. SF36 = 36-Item Short Form Survey

Among the variables related to the quality of life, in the direct path, quality of life had the highest negative causal relationship with DASS-21 score (B = − 0.26), with a one-unit increase in the score of DASS-21, quality of life was reduced by 0.26 units. Among the variables with a significant causal relationship with quality of life in the indirect path, socioeconomic status had the highest positive relationship with quality of life (B = 0.02). In contrast, the mean duration of using social networks (B ≈ − 0.07) and internet emotional relationships (B ≈ − 0.09) had the highest negative relationship with this index, and one-unit increase in the score of Internet emotional relationships and the mean duration of using social networks reduced the quality of life score by 0.09 and 0.07 units, respectively. Furthermore, a one-unit increase in socioeconomic score increased the quality of life score by (0.02) units. These findings are illustrated in Table 4 and Fig. 2.

**Table 4:** Direct and indirect effects of social networks and Internet emotional relationships with mental health and quality of life

		Standard B			Unstandardized β		
		Direct effects	Indirect effects	Total Effect	Direct effects	Indirect effects	Total Effect
mental health (DASS21)	EDU	0.12^a^	0.36	0.12^a^	1.17^a^	0.034	1.17^a^
	TUS	0.08	0.288	0.11	0.55	0.19	0.74
	SES	− 0.09^a^	0.0012	− 0.09^a^	− 0.33^a^	0.005	0.33^a^
	INT	0.22^a^	0.013	0.22^a^	0.17^a^	0.01	0.17^a^
	SOM	0.06	–	0.06	0.09	–	0.09
Quality of life (SF-36)	EDU	0.04	−0.03^a^	−0.03^a^	0.18	− 0.14^a^	− 0.14^a^
	TUS	0.04	−0.067^a^	− 0.067^a^	0.13	−0.213^a^	− 0.213^a^
	SES	−0.06	0.0234^a^	0.0234^a^	−0.09	0.039^a^	0.039^a^
	INT	0.02	−0.089^a^	−0.089^a^	0.008	−0.032^a^	− 0.032^a^
	SOM	−0.14^a^	–	−0.14^a^	− 0.1^a^	**–**	−0.1^a^
	DASS	−0.26^a^	–	−0.26^a^	− 0.12^a^	–	−0.12^a^

*EDU* Education, *TUS* Average time of use of the Internet, *INT* Internet Emotional Relationships, *SOM* Social Network Score, *DASS* Depression, Anxiety and Stress Scale, *SES* Socioeconomic Status, *SF36* 36-Item Short-Form Health Survey

^a^significant

The fitness of model indices showed that the model has a good fit and is highly compatible, and the adjusted relationships of the variables based on the conceptual model are rational. Accordingly, there was no significant difference between the fitted and conceptual models (Table 5).

**Table 5:** Model Fitting Indicators

RMSEA (root mean squared error of approximation)	NFI (Bentler-Bonett Normed fit index)	GFI (Goodness of fit index)	CFI (comparative fit index)	X^2^/df	df	X^2^
0000	0.99	1	1	1.06	3	3.18

**Fig. 3:**
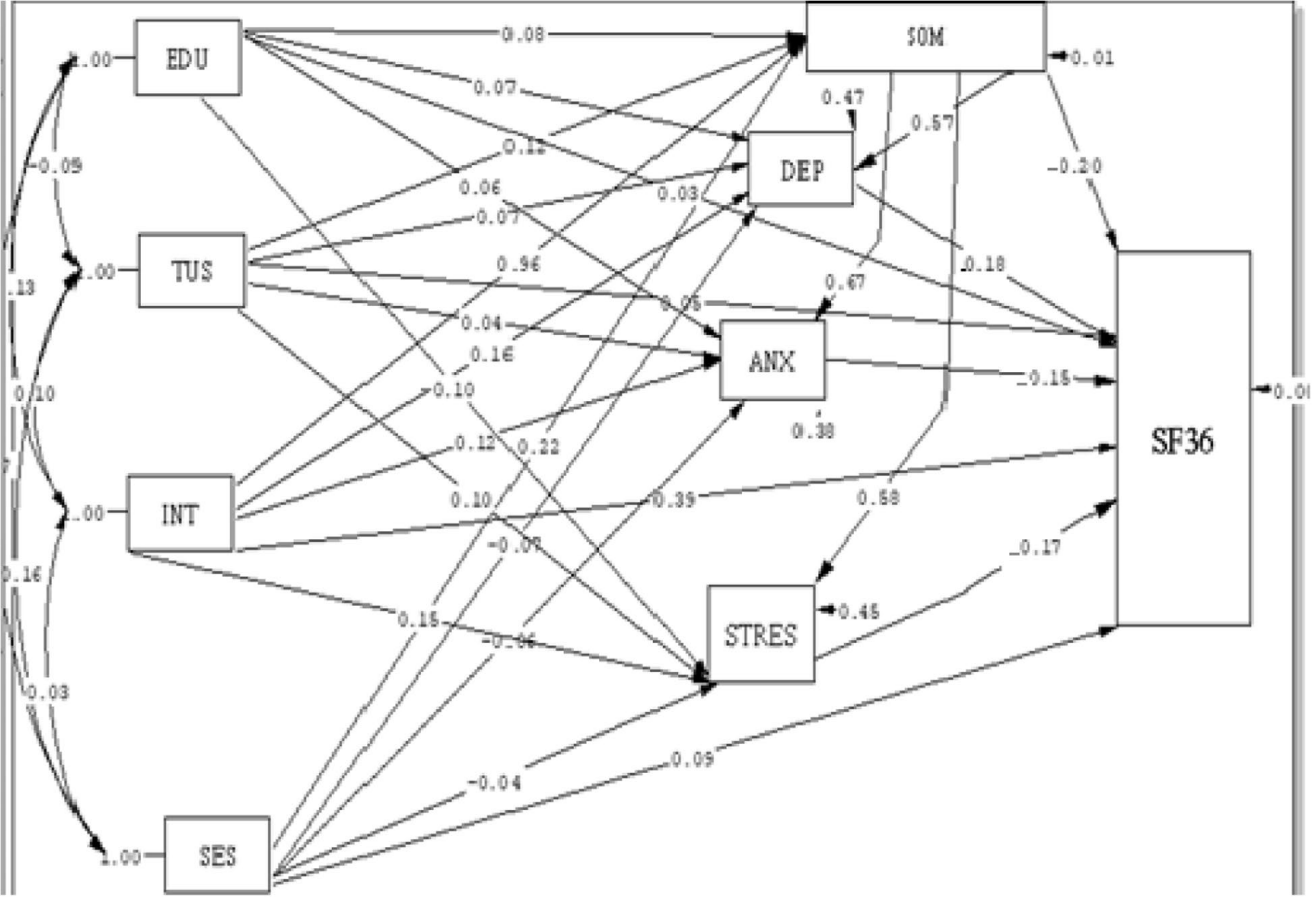
Full Empirical Path Model between Social networks and Internet emotional relationships on Depression, Anxiety, Stress and quality of life in students. Single-headed arrow means regression coefficient, Standardized Beta. Two-headed arrow means correlation. EDU = Education. TUS = Average time of use of the Internet, INT = Internet Emotional Relationships. SOM = Social Network Score, DEP = Depression, ANX = Anxiety and STRES=Stress, SES = Socioeconomic Status, SF36 = 36-Item Short-Form Health Survey

Figure 3 illustrates the path analysis of the empirical path model between social networks and “internet emotional relationships on Depression, Anxiety, Stress, and quality of life in students. Among the variables that had a significant causal relationship with anxiety, in the direct path, Social Network Score (B = 0.67) and in the indirect path, internet emotional relationships (B = 0.64) had the most positive causal relationship with anxiety. The only variable with a significant causal relationship with stress was education (B = − 0.10). Moreover, this model did not see any significant causal relationship between variables and depression.

Among the variables with a significant causal relationship with quality of life, in the direct path, depression (B = − 0.18), stress (B = − 0.17), and anxiety (B = − 0.15) had a negative significant causal relationship with quality of life. Internet emotional relationships had the most positive causal relationship with quality of life (B ≈ 0.20) In Both ways (direct and indirect paths). (Table 6).

**Table 6:** Direct and indirect effects of social networks and Internet emotional relationships with Dominate of DASS21(Stress, Depression, Anxiety) and quality of life

			Direct effects	Indirect effects	Total Effect
DASS 21	Depression	EDU	0.07	0.045	0.11
		TUS	0.07	0.068	0.138
		SES	−0.07	0.125	0.055
		INT	0.16	0.54	0.7
		SOM	0.57	–	0.57
	Anxiety	EDU	0.06	0.053^a^	0.053^a^
		TUS	0.04	0.08^a^	0.08^a^
		SES	−0.06	0.14^a^	0.14^a^
		INT	0.12	0.64^a^	0.64^a^
		SOM	0.67^a^	–	0.67^a^
	Stress	EDU	−0.1^a^	0.046	− 0.1^a^
		TUS	0.1	0.06	0.16
		SES	−0.04	0.12	0.08
		INT	0.15	0.55	0.7
		SOM	0.58	–	0.58
Quality of life (SF-36)		EDU	0.03^a^	0.001^a^	0.031^a^
		TUS	0.05^a^	−0.024^a^	0.026^a^
		SES	0.09^a^	−0.044^a^	0.046^a^
		INT	0.39^a^	−0.192^a^	0.198^a^
		SOM	−0.2^a^	–	−0.2^a^
		Depression	−0.18^a^	–	−0.18^a^
		Anxiety	−0.15^a^	–	−0.15^a^
		Stress	−0.17^a^	–	−0.17^a^

*EDU* Education, *TUS* Average time of use of the Internet, *INT* Internet Emotional Relationships, *SOM* Social Network Score, *SES* Socioeconomic Status, *SF36* 36-Item Short-Form Health Survey

^a^significant

## Discussion

Students experience a unique transition from high school to adulthood in the course of their education [32]; which marks the beginning of a major period of psychological and social development that can have different impacts on students’ health and life [33]. In this study, we assessed the association of the selected variable on mental health and quality of life separately.

According to the results obtained, among the variables with a significant causal relationship with mental health in the students, socioeconomic status had the highest direct negative relationship, meaning lower SES scores were associated with higher DASS scores. This finding is in line with the results obtained by Silva et al., who found in their review study of 2004 to 2014 that there is an independent relationship between mental health and socioeconomic status [34]. In a cohort study conducted on adolescents, Reiss et al. (2020) found that socioeconomic status is an independent predictor of mental health problems. Adolescents with bad socioeconomic conditions experience various stressful situations in their life, thus exposing them to mental problems or exacerbations [35]. The results of many studies indicate that there are more stressful situations in the life of families with a lower socioeconomic status, such as parents’ mental illness or accidents, severe financial crisis, job loss, academic problems and etc. compared to other groups, which can contribute to the development of mental disorders [34–36].

In the present study, Internet emotional relationships had a positive causal relationship with a higher DASS-21 score in the direct path. In other words, students’ depressive and anxiety symptoms and stress levels increased as their score of internet emotional relationships increased. This finding can be the consequence of the introduction of new communication technologies, including the changes in the type and method of communication between the two sexes, mutual emotional interactions, and different patterns of intimacy [37]. Researchers have found conflicting results about these forms of friendship. Online friendships are defined as fragile relationships with less attachment, commitment, and self-disclosure that deter people from pursuing social activities and impair their development of social relationships. However, online friendships are regarded as an alternative to positive experiences and beneficial and stable relationships [38]. Researchers have found that feeling left out of the family and society and the absence of a robust social network like friends persuade people to pursue online relationships to make up for their emotional deficiencies, thus resulting in different levels of internet addiction [39]. In a study conducted on university students, Alavi et al. found that there is a significant relationship between mental disorders, including depression and internet dependencies, which is in line with the present findings [40] yet, contradicts the results of the study by Hachebi (2001), who argued that social media provides a good opportunity for people and can affect their mental health [41]. These contradictions can be caused by the method and level of use of social networks and the level of emotional-social dependence due to cultural-social limitations. The rise in these contradictions is alarming not only for policymakers and civil community groups but also for the citizens [42].

High DASS-21 scores had a negative, causal association with quality of life, such that an increase in depressive, anxiety, and stress scores, reduced the students’ quality of life. The present findings agree with the results of many studies, including a qualitative systematic review study by Connell et al. (2012), which found that a good quality of life is correlated with a good sense of control, a positive self-image, and hope and optimism, and conversely, a low quality of life is correlated with mental disorders, anxiety and poor self-esteem [43]. In another study conducted on university students, Jenkins et al. (2020) confirmed the effect of mental disturbances on quality of life and argued that students with anxiety, depression, or other mental disturbances had a lower quality of life [44].

Other factors with a significant causal relationship with quality of life included the average time spent on the internet and online emotional relationships, which had the greatest negative relationship with quality of life. Some researchers have found that internet use is significantly associated with reduced communication with friends and social networks, which causes further loneliness and a decrease in some aspects of quality of life [45]. In a study conducted on nursing students, Ragheb et al. (2018) found that inappropriate use of the internet adversely affects the students’ quality of life. They believed that excessive use of the internet was a way for the students to run away from the pressures of real-life or parental conflicts [46], which agrees with the present findings.

## Limitation

This study was conducted in the COVID-19 Pandemic. Hence, we had some limitations to access students, and another limitation was the use of questionnaires, in which the given answers may be biased. Moreover, the cross-sectional nature of the study limited us in assessing the temporal relationship of exposure and outcomes. Furthermore, a larger sample size is needed to make sure the representativeness of the sample.

## Conclusion

According to the results, using the internet and virtual networks, online emotional relationships, and lower socioeconomic status are associated with higher DASS-21 scores (depression, anxiety and stress) and reduced quality of life in students. Since students are essential for the future of every country, it is necessary for policymakers to further address this group and their concerns. The researchers recommend further training of the public on proper internet use and virtual networks and their role in improving the quality of life.

## Data Availability

The data that support the findings of this study are available from the corresponding author upon reasonable request.

## Abbreviations

EDU: Education
TUS: Average time of use of the Internet
INT: Internet Emotional Relationships
SOM: Social Network Score
DASS: Depression, Anxiety and Stress Scale
SES: Socioeconomic Status
SF36: 36-Item Short-Form Survey
